# Prevalence, intensity, and risk factors of schistosomiasis and intestinal parasitic infections among primary school children in northern Uganda: Implications for public health interventions

**DOI:** 10.1371/journal.pntd.0013827

**Published:** 2025-12-17

**Authors:** John Paul Byagamy, Robert Opiro, Harriet Angwech, Margaret Nyafwono, Geoffrey Maxwell Malinga, Richard Echodu, Emmanuel Igwaro Odongo-Aginya

**Affiliations:** 1 Department of Environment and Natural Resources Management, Faculty of Agriculture and Environment, Gulu University, Gulu, Uganda; 2 Department of Biology, Faculty of Science, Gulu University, Gulu, Uganda; 3 Department of Microbiology and Immunology, Faculty of Medicine, Gulu University, Gulu, Uganda; Institute of Cytology and Genetics SB RAS: FIC Institut citologii i genetiki Sibirskogo otdelenia Rossijskoj akademii nauk, RUSSIAN FEDERATION

## Abstract

**Background:**

Schistosomiasis and intestinal parasitic infections remain major public health concerns in Uganda, particularly among school-aged children, where they contribute to anemia, malnutrition, and poor cognitive development. The Lango sub-region of Northern Uganda is endemic for *Schistosoma mansoni*, yet epidemiological data remain scarce. We conducted this study to determine the prevalence, intensity, and risk factors of infection to guide targeted control strategies.

**Methodology/principal findings:**

We conducted a cross-sectional study between January and March 2023, involving 802 primary school children from randomly selected schools in Lira District, Lira City, and Kole District. Stool samples were examined using the Odongo-Aginya method to detect *Schistosoma mansoni* and intestinal parasites. Urine samples were screened using the Point-of-Care Circulating Cathodic Antigen (POC-CCA) test for *Schistosoma mansoni* and urine filtration for *Schistosoma haematobium*. Data on potential risk factors were collected via structured interviews and analyzed using logistic regression in SPSS version 25.0. The overall prevalence of schistosomiasis was 34.5% (*S. mansoni*), with light (11.6%), moderate (5.4%), and heavy (2.9%) infection intensities. Other intestinal parasites were detected in 20.3% of participants, including *Ascaris lumbricoides* (11.6%) and hookworms (6.4%). Children in P.3 (OR = 3.19, 95% CI: 1.67–6.08), P.4 (OR = 3.22, 95% CI: 1.71–6.08), and P.6 (OR = 2.62, 95% CI: 1.26–5.47) had significantly higher odds of *S. mansoni* infection compared to P.7, with Ayara pupils most at risk (OR = 35.05, 95% CI: 9.30–132.14). Elevated risks occurred in Apedi, Aberdyangotoo, and Bala schools, while pupils in Kole (OR = 0.46, 95% CI: 0.33–0.63) and Lira City (OR = 0.48, 95% CI: 0.20–1.14) had reduced odds. Low paternal education increased risk, whereas recent praziquantel treatment markedly reduced infection (OR = 0.10, p = 0.027).

**Conclusions/significance:**

The study highlights the persistent and focal nature of schistosomiasis transmission in northern Uganda, shaped by school and district-level factors, socioeconomic disparities, and irregular MDA implementation. Strengthening and sustaining praziquantel distribution, expanding WASH infrastructure, and introducing targeted, school-focused interventions are essential to reduce reinfection and sustain control efforts. Addressing these factors is critical for Uganda to progress toward schistosomiasis elimination goals.

## Author summary

Schistosomiasis and intestinal parasitic infections are significant public health challenges, especially in low-resource settings like Northern Uganda. These infections can lead to malnutrition, anemia, and impaired cognitive development, particularly in school-aged children. In this study, we investigated the prevalence, intensity, and risk factors for these infections among primary school children in the Lango sub-region of Northern Uganda. We examined stool and urine samples from over 800 children and found that more than one-third were infected with *Schistosoma mansoni*, the parasite responsible for intestinal schistosomiasis. Additionally, we identified other intestinal parasites, such as *Ascaris lumbricoides* (roundworms) and *hookworms*. Our findings show that school and district-level factors, socioeconomic disparities, low parental education levels, and irregular MDA implementation were linked to a higher risk of infection. However, children who had recently received treatment with praziquantel, the main drug used for schistosomiasis, were less likely to be infected. These results highlight the urgent need for expanded treatment programs, improved sanitation, and targeted health education efforts to reduce the burden of parasitic infections in Ugandan schoolchildren. By highlighting key risk factors, this study offers important evidence to inform policymakers and public health practitioners in developing targeted interventions, thereby supporting national and global efforts to accelerate schistosomiasis elimination.

## Introduction

Schistosomiasis and intestinal parasitic infections are among the most prevalent neglected tropical diseases globally [1], disproportionately affecting populations in low- and middle-income countries [2]. These infections, caused by trematodes and soil-transmitted helminths (STH), respectively, contribute significantly to morbidity, particularly in children, due to their impact on growth, cognitive development, and overall health [3,4]. In sub-Saharan Africa, where sanitation and access to clean water are limited, schistosomiasis and intestinal parasitic infections remain pervasive public health challenges [5–7].

In Uganda, *Schistosoma mansoni* is endemic in many regions, including the Lango sub-region in Northern Uganda [8,9]. However, it’s only in the Lango sub-region known to harbor both *S. mansoni* and *S. haematobium* [10–12]. The disease is primarily transmitted through contact with freshwater bodies contaminated with cercariae released by infected snail hosts [13,14]. Children are especially vulnerable due to frequent water contact activities, such as swimming, washing clothes/dishes, fishing, crossing water bodies and fetching water, often in infested environments [15–17]. Beyond schistosomiasis, soil-transmitted helminths, including *Ascaris lumbricoides*, *Hookworms*, and *Trichuris trichiura*, are widespread, perpetuating cycles of poverty and illness [18,19].

Currently, the control measures for Schistosomiasis and soil-transmitted helminths control in Uganda is predominantly based on mass drug administration (MDA) campaigns using Praziquantel and Albendazole, which are supported by the Ministry of Health and international partners [20,21] and carried out annually in endemic areas [9]. However, the effectiveness of these programs is contingent on consistent drug distribution, compliance, adequate coverage, and complementary interventions such as enhanced water, sanitation, and hygiene (WASH) activities [21,22]. Previous impact assessments have shown that, while MDA has significantly reduced schistosomiasis prevalence in some regions, reinfection is nevertheless common due to continued exposure hazards and inadequacies in WASH infrastructure [20]. Environmental factors, socio-economic disparities, and a lack of community-level data further hinder effective control strategies [22,23]. Several studies have reported varying prevalence rates of schistosomiasis and intestinal parasitic infections across Uganda [8,20,22]. For instance, the prevalence of *S. mansoni* in school-aged children ranges from 20% to 60%, depending on the region [14,24,25]. However, there is a paucity of data specific to the Lango sub-region, where unique environmental and socio-economic conditions may influence disease distribution and risk factors [10,17,26]. This study aimed to bridge the knowledge gap by determining the prevalence, intensity, and risk factors associated with schistosomiasis and intestinal parasitic infections among primary school children in the Lango sub-region. The findings aim to inform local and national policy-makers and stakeholders on targeted intervention strategies to reduce the disease burden in Northern Uganda.

## Methods and materials

### Ethics approval and consent to participate

Ethical approval for this study was obtained from the Gulu University Research and Ethics Committee (GUREC-2022–323). Additional approval was obtained from the Uganda National Council for Science and Technology (UNCST-HS2571ES). Written informed consent was obtained from parents or legal guardians of participating children, and assent was obtained from children aged 8 years and above. All methods were performed in accordance with relevant guidelines and regulations.

### Study area

**Fig 1:**
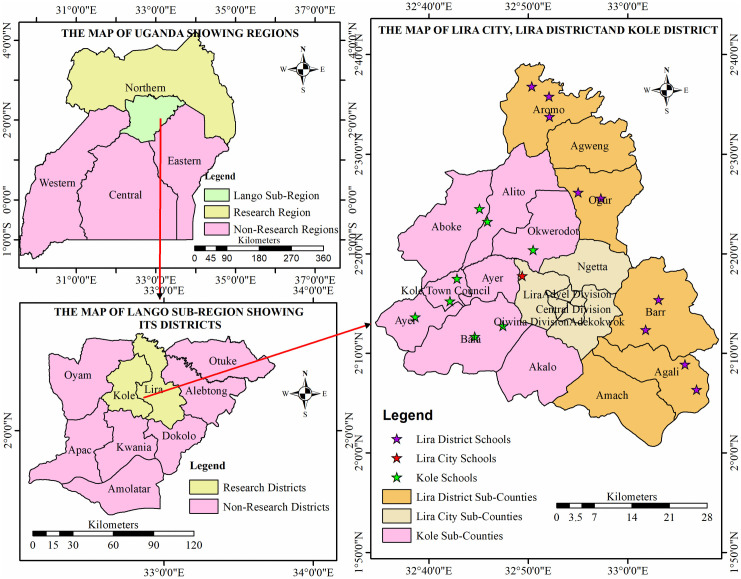
Study sites showing schools surveyed for schistosomiasis and intestinal parasitic infections in Lira City, Lira and Kole districts, northern Uganda. Base map was created in ArcMap 10.5 using administrative boundary shape files from DIVA-GIS (https://diva-gis.org/data.html; source: GADM, https://gadm.org/license.html).

This study was conducted in the Lango sub-region, Northern Uganda (Fig 1), specifically targeting three administrative areas: Lira District, Lira City, and Kole District [27]. These districts are among the nine districts in the Lango sub-region, predominately inhabited by the Lango tribe with a total population of 726,675 [27]. The number of primary schools in the study area is 317 schools [27]. The main economic activities are commercial and subsistence farming, small-scale fishing, retail, and wholesale business. These regions are characterized by a tropical climate, seasonal rainfall, and reliance on freshwater sources for domestic and recreational activities, which predispose residents to schistosomiasis transmission [28]. The study area is part of Uganda’s national schistosomiasis control program, which implements an annual Mass Drug Administration (MDA) with praziquantel [9]. In Lira city, Lira district and Kole district, the Ministry of Health also implements child health days plus (CHD+), during which school-aged children (5–16 years) receive vitamin A supplementation and deworming with Albendazole or Mebendazole, and, in schistosomiasis endemic communities, praziquantel is co-administered through school-based MDA [9]. However, the timing and coverage of MDA in these districts have been inconsistent, with some schools or communities experiencing delays or missed distributions [29]. This irregularity likely contributed to the low number of children who reported receiving praziquantel within the three months preceding this study (Table 3), despite residing in an MDA-targeted area.

**Table 3:** Prevalence of *Schistosoma mansoni* among school children.

		Infection status				
Variable	Category	Negative (%)	Positive (%)	χ^2^	df	p-value
District	Kole District	288 (54.9)	110 (39.7)	27.64	2	**< 0.001** ^*^
	Lira City	35 (6.7)	8 (2.9)			
	Lira District	202 (38.5)	159 (57.4)			
Gender	Female	242 (46.1)	119 (43.0)	0.72	1	0.396
	Male	283 (53.9)	158 (57.0)			
Age group	5-7	14 (2.7)	9 (3.2)	4.02	3	0.259
	8-10	82 (15.6)	47 (17.0)			
	11-13	267 (50.9)	154 (55.6)			
	14-16	162 (30.9)	67 (24.2)			
Class	P1	34 (6.5)	13 (4.7)	31.678	6	**< 0.001** ^*^
	P2	29 (5.5)	8 (2.9)			
	P3	113 (21.5)	79 (28.5)			
	P4	114 (21.7)	79 (28.5)			
	P5	114 (21.7)	39 (14.1)			
	P6	47 (9.0)	28 (10.1)			
	P7	74 (14.1)	18 (6.5)			
School	Aberdyangotoo	25 (4.8)	13 (4.7)	140.112	19	**< 0.001** ^*^
	Abilonino	37 (7.0)	3 (1.1)			
	Abolet	14 (2.7)	25 (9.0)			
	Acutkumu	33 (6.3)	14 (5.1)			
	Akano	19 (3.6)	23 (8.3)			
	Akore	18 (3.4)	22 (7.9)			
	Apedi	26 (5.0)	15 (5.4)			
	Ayara	8 (1.5)	33 (11.9)			
	Ayer	32 (6.1)	10 (3.6)			
	Bala	28 (5.3)	14 (5.1)			
	Lwala	35 (6.7)	7 (2.5)			
	Obot	28 (5.3)	7 (2.5)			
	Obuto	34 (6.5)	2 (0.7)			
	Ocamoyang	27 (5.1)	13 (4.7)			
	Ogur	26 (5.0)	11 (4.0)			
	Oketkwer	22 (4.2)	19 (6.9)			
	Okwerodot	28 (5.3)	9 (3.2)			
	Okwor	35 (6.7)	4 (1.4)			
	Ororo	15 (2.9)	25 (9.0)			
	Teokole	35 (6.7)	8 (2.9)			
Sub county	Aboke	26 (5.0)	15 (5.4)	42.6	8	**< 0.001** ^*^
	Agali	42 (8.0)	38 (13.7)			
	Aromo	73 (13.9)	55 (19.9)			
	Ayer	104 (19.8)	17 (6.1)			
	Bala	53 (10.1)	27 (9.7)			
	Barr	42 (8.0)	32 (11.6)			
	Lira	35 (6.7)	8 (2.9)			
	Ogur	45 (8.6)	34 (12.3)			
	Okwerodot	105 (20.0)	51 (18.4)			
Water source for bathing	Bore hole	330 (62.9)	154 (55.6)	10.677	7	0.153
	Dam	2 (0.4)	1 (0.4)			
	Piped water	56 (10.7)	39 (14.1)			
	Shallow Well	1 (0.2)	0			
	Spring	24 (4.6)	19 (6.9)			
	Stream	3 (0.6)	6 (2.2)			
	Tap water	5 (1.0)	1 (0.4)			
	Well	104 (19.8)	57 (20.6)			
Water source for washing	Bore hole	326 (62.1)	151 (54.5)	15.092	7	**0.035** ^*^
	Dam	2 (0.4)	0			
	Piped water	55 (10.5)	38 (13.7)			
	Shallow Well	1 (0.2)	0			
	Spring	23 (4.4)	19 (6.9)			
	Stream	3 (0.6)	8 (2.9)			
	Tap water	5 (1.0)	1 (0.4)			
	Well	110 (21.0)	60 (21.7)			
Father’s education	Illiterate	63 (12.0)	28 (10.1)	9.742	4	**0.045** ^*^
	Primary	356 (67.8)	177 (63.9)			
	Secondary	70 (13.3)	56 (20.2)			
	Tertiary	32 (6.1)	11 (4.0)			
	University	4 (0.8)	5 (1.8)			
Praziquantel administered 3 months ago	No	503 (95.8)	276 (99.6)	9.546	1	**0.02** ^*^
	Yes	22 (4.2)	1 (0.4)			

### Study design

A cross-sectional study design was employed to assess the prevalence, intensity, and risk factors associated with schistosomiasis and intestinal parasitic infections among school-aged children. Data collection occurred between January and March 2023.

### Study population

The study targeted primary school children aged 5–16 years enrolled in Primary 1–7. Participants were stratified into districts, sub-counties, schools, and classes (primary 1–7), and a representative number proportionate to the school population was selected randomly from each district, school, and class and screened for intestinal Schistosomiasis and other intestinal parasites. This cohort was chosen because school-aged children are most at risk of schistosomiasis and intestinal parasitic infections due to frequent water contact and poor hygiene practices.

### Inclusion and exclusion criteria

Children aged 5–16 years who attended selected primary schools in Lira District, Lira City, and Kole District and had resided there for at least one year, written informed consent obtained from parents or guardians for minors, and assent from children aged 8 years and above were included. Age categories were defined as 5–7, 8–10, 11–13, and 14–16 years old to match with developmental stages and schooling patterns in Uganda. These categories reflect key transitions in early childhood, middle childhood, early adolescence, and late adolescence, which may influence exposure risks, immunity development, and behavioral factors associated with schistosomiasis transmission [30]. At the school level, schools in which CHD+ school-based MDA with praziquantel had been conducted immediately before recruitment were excluded from the sampling frame, because recent mass praziquantel administration at the school level was considered incompatible with the study objective of assessing current burden under usual programmatic conditions.

### Sample size determination

The sample size was calculated using the formula for cross-sectional studies [31].

Where: N = Z^2^ × PQ/d^2.^ Where, N = the desired sample size, P = estimated prevalence, estimated to be 50%, Q = percentage of people not infected (1-p), d = degree of precision required, usually set at 0.05, Z = confidence limit at 95% interval (1.96). Adjusting for a 10% non-response rate, the final sample size was 802 participants.

### Sampling methods

A multistage sampling technique was used to select participants. First, a sampling frame of 317 primary schools from Lira district, Lira city, and Kole district was compiled [27]. Schools were stratified by administrative units, and the number of schools to be included from each administrative unit was allocated proportionally to the total number of schools in the administrative units. Within each administrative unit stratum, individual schools were then selected using computer-generated random numbers, yielding a total of 20 schools. However, prior to the recruitment of children, the Ministry of Health conducted CHD+ school-based MDA [9], which included praziquantel, in almost all of the initially selected Lira city schools. Because recent school-wide praziquantel MDA was an exclusion criterion at the school level, these Lira city schools were no longer eligible for inclusion. Te-Okole primary school, the only Lira city school that had not yet received MDA, remained eligible. To maintain the planned number of 20 schools, the ineligible Lira city primary schools were replaced with additional randomly selected schools from Lira and Kole districts. Within each selected school, participants were stratified by grade (P1-P7), and simple random sampling was employed to select children proportionate to the school’s population. Accordingly, 802 school children aged 5–16 years were randomly sampled from 20 primary schools across Lira district, Lira City, and Kole Districts, respectively.

### Stool and urine examinations

Only children attending school in the selected primary schools during the study period who willingly volunteered to participate in the study were provided with stool containers, well labelled with the participant’s code, laboratory number, date, and time of sample collection. Each participant was instructed to collect an equivalent of 10gm of stool between 8 and 10 am and deliver it to the laboratory within one hour after the specimen collection. The stool specimens were processed using the Odongo –Aginya method (compound stain: consisting of 5% eosin yellow in 10% formalin mixed 1:1 with 7.5% Nigrosin in 10% formalin) [32]. Cellophane cover slips cut in 25 × 40 mm pieces were pre-soaked in 50% glycerine [32].

Stool specimens were strained through a sieve, cleaned, and placed on a microscope slide through a template measuring 41.7mg. A compound stain was added, and a cellophane cover slip was blotted out. The slide was then examined microscopically to quantify Schistosome eggs in faeces [32]. Specimen processing and examination were done at the Lira Regional Referral Hospital (LRRH) Laboratory and duplicate slides were read by two qualified technicians and re-examined by a senior laboratory technologist for quality control [32]. Urine samples were collected in sterile, well-labeled containers with participant’s ID codes to prevent contamination. Participants were provided with detailed instructions on proper sample collection to ensure the integrity of the specimens for accurate diagnostic testing. 20–30ml of freshly voided urine was collected from each participant between 10 am to 2 pm, transported in a cold chain and tested within one hour of collection at LRRH laboratory following the standard protocols by Wilson et al. [33]. The study used a commercial antigen test kit called Point-of-Care Circulating Cathodic Antigen assay (POC-CCA) to measure the CCA levels of juvenile and adult *S. mansoni* in the urine [34]. The test was performed and reported according to the manufacturer’s instructions. The tests were interpreted by lab technicians independently to accurately assess the infection prevalence [34]. The urine filtration technique was also employed to examine and quantify *S. haematobium* in urine [35] by experienced laboratory technicians in the LRRH laboratory. A 10 ml syringe with a Swinney filter holder of 13mm diameter and polycarbonate membrane filter was employed to recover the eggs of *S. haematobium* in urine and placed on a glass slide, examined using a light microscope at ×40 objective lens [35]. Samples found to have eggs were recorded as positive and the number of eggs counted to determine the intensity. Those found to be positive during the study were treated with praziquantel at a dose of 40 mg/kg body weight free of charge [36]. Albendazole 400 mg was also administered as a single dose to all participating children to address soil-transmitted helminth infections. This treatment was provided in accordance with the Uganda national guidelines for the control of schistosomiasis and intestinal parasitic infections [37].

### Risk factors and demographic data collection

Data on potential risk factors, including water contact activities, sanitation practices, parental education, and prior praziquantel treatment in the last three months, were collected through structured interviews using pretested standardized questionnaire forms (S2 File). Demographic data, such as age, gender, and school, were also recorded. Trained field assistants interviewed the study participants.

### Data analysis

Data were analyzed using IBM SPSS for windows version 25.0 [38]. Descriptive statistics were used to calculate the prevalence and intensity of infections. The intensity of *S. mansoni* infection was calculated using WHO guidelines [35], categorized as light (1–99 epg), moderate (100–399 epg), and heavy (>400 epg). Associations between potential risk factors and infection status was evaluated first using chi-square tests. Possible risk factors from the univariate analysis (all with a P < 0.2) were then included in backward stepwise logistic regression model. A backward stepwise multivariable logistic regression was used to determine the risk factors associated with *S. mansoni* infection among children of school going age. At each step, variables were excluded based on p-values and a p-value threshold of 0.2 [39] was used to set a limit on the total number of variables included in the final multivariate logistic regression model. Finally, variables with a p-value of ≤ 0.05 in multivariate analysis were considered as statistically significant and odds ratio with 95% CI was considered to see the association. The Hosmer and Lemeshow goodness of fit tests were done to check the model’s fitness (S1 File).

## Results

### Socio-demographic characteristics of study participants

The study included participants from three districts in the Lango sub-region. The highest proportion of participants came from Kole District (n = 398, 49.6%), followed by Lira District (n = 361, 45%), with a smaller representation from Lira City (n = 43, 5.4%). Gender distribution showed a higher percentage of males (n = 441, 55%) than females (n = 361, 45%). The mean age was 12.25, SD ± 2.113. The majority of participants were aged between 11–13 years (n = 421,52.5%), followed by those aged 14–16 years (n = 229, 28.6%). Smaller proportions were observed in the 8–10 years (n = 129, 16.1%) and 5–7 years (n = 23, 2.9%) age groups (Table 1).

**Table 1:** Sociodemographic characteristics of study participants in the Lango sub-region (n = 802).

Variables	Categories	Frequency (n)	Percentage (%)
**District**	Kole District	398	49.6
	Lira City	43	5.4
	Lira District	361	45
**Gender**			
	Female	361	45
	Male	441	55
**Age group (years)**			
	5-7	23	2.9
	8-10	129	16.1
	11-13	421	52.5
	14-16	229	28.6
**Class**			
	P 1	47	5.9
	P 2	37	4.6
	P 3	192	23.9
	P 4	206	25.7
	P 5	153	19.1
	P 6	75	9.4
	P 7	92	11.5
**Sub-county**			
	Aboke	41	5.1
	Agali	80	10
	Aromo	128	16
	Ayer	121	15.1
	Bala	80	10
	Barr	74	9.2
	Lira	43	5.4
	Ogur	79	9.9
	Okwerodot	156	19.5

Participants were distributed across different primary school classes, with the largest groups in P4 (n = 206, 25.7%) and P3 (n = 192, 23.9%). Smaller percentages were seen in classes P1 (n = 47, 5.9%) and P2 (n = 37, 4.6%), while P5, P6, and P7 accounted for (n = 153, 19.1%), (n = 75, 9.4%), and (n = 92, 11.5%), respectively (Table 1). The sub-county distribution showed the highest representation from Okwerodot (n = 156, 19.5%), followed by Aromo (n = 128, 16%) and Ayer (n = 121, 15.1%). Other sub-counties had lower proportions, such as Aboke (n = 41, 5.1%) and Lira (n = 43, 5.4%), Table 1.

### Overall prevalence of parasites identified

The study identified various parasitic infections among participants, with *Schistosoma mansoni* being the most prevalent, affecting (n = 277, 34.5%) of the participants. Other notable parasites included *Entamoeba coli* (n = 94, 11.7%) and *Ascaris lumbricoides* (n = 93, 11.6%).

Lower prevalence rates were observed for *Hookworms* (n = 51, 6.4%), *Enterobius vermicularis* (n = 26, 3.2%), and *Trichuris trichura* (n = 12, 1.5%). Rare cases were recorded for *Fasciola species* (n = 11, 1.4%), *Entamoeba histolytica* (n = 10, 1.2%), and *Taenia species* (n = 7, 0.9%).

Very low prevalence (n = 1, 0.1%) was found for *Giardia lamblia*, *Paragonimus westermani*, *Strongyloides stercolaris*, and *Blastocystis spp.* (Table 2). However, *S. haematobium* was not detected in all the urine samples examined.

**Table 2:** Overall prevalence of parasites identified.

Parasites	No. Positive (n)	Prevalence (%)
*Schistosoma mansoni*	277	34.5
*Entamoeba coli*	94	11.7
*Ascaris lumbricoides*	93	11.6
*Hookworms*	51	6.4
*Enterobius vermicularis*	26	3.2
*Trichuris trichura*	12	1.5
*Fasciola species*	11	1.4
*Entamoeba histolytica*	10	1.2
*Taenia species*	7	0.9
*Blastocystis spp.*	1	0.1
*Giardia lamblia*	1	0.1
*Paragonimus westermani*	1	0.1
*Strongyloides stercolaris*	1	0.1

### Prevalence of *Schistosoma mansoni* among school children

The study evaluated the prevalence of *Schistosoma mansoni* infection based on sociodemographic, educational, and behavioral variables. The prevalence was highest in Lira District (n = 159, 57.4%) and lowest in Lira City (n = 8, 2.9%), with Kole District showing (n = 110, 39.7%) positivity (p < 0.001). No significant difference was observed between males (n = 158, 57.0%) and females (n = 119, 43.0%) (p = 0.396), (Table 3). The highest prevalence was in the 11–13 age group (n = 154, 55.6%), but no significant association was found (p = 0.259). Participants in the P3 and P4 classes had the highest prevalence (n = 79, 28.5%) each, while P2 and P1 had the lowest (p < 0.001). The sub-counties of Aromo (n = 55, 19.9%) and Okwerodot (n = 51, 18.4%) recorded the highest prevalence (chi-square χ2 = 42.6, p < 0.001) (Table 3). There was no significant association between water source for bathing and infection status (χ2 = 10.68, p = 0.153). However, a significant association was observed between water sources used for washing and *S. mansoni* prevalence (χ2 = 15.09, p = 0.035). Children who reported using streams (n = 8, 2.9%) and wells (n = 60, 21.7%) had a higher prevalence of infection compared to those using other water sources. Lower prevalence was observed among participants with fathers having tertiary or university education (χ2 = 9.74, p = 0.045), while participants who received praziquantel within the last three months had significantly lower prevalence (χ2 = 9.55, p = 0.02), (Table 3).

### Intensity of infection with *Schistosoma mansoni* among the study participants

The study assessed the infection intensity of *S. mansoni* infection among participants using eggs per gram (epg) of stool, categorized as light (1–99 epg), moderate (100–399 epg), and heavy (>400 epg). Overall infection intensity was light (n = 93, 11.6%), moderate (n = 43, 5.4%), and heavy (n = 23, 2.9%). In Kole District, (n = 28, 7.04%) had light infections, (n = 23, 5.78%) moderate, and (n = 9, 2.26%) heavy infections. Lira City recorded only light infections (n = 6, 13.95%) with no moderate or heavy cases. In Lira District, (n = 59, 16.34%) had light infections, (n = 20, 5.54%) moderate, and (n = 14, 3.88%) heavy (Table 4).

**Table 4:** Intensity of infection with *Schistosoma mansoni* among study participants.

			Intensity of infection		
Variables	Category	Participants (n)	Light (1–99 epg), n(%)	Moderate (100–399 epg), n(%)	Heavy (> 400 epg), n(%)
District					
	Kole District	398	28 (7.04)	23 (5.78)	9 (2.26)
	Lira City	43	6 (13.95)	0	0
	Lira District	361	59 (16.34)	20 (5.54)	14 (3.88)
Gender					
	Female	361	39 (10.80)	16 (4.43)	7 (1.94)
	Male	441	54 (12.24)	27 (6.12)	16 (3.63)
Age group					
	5-7	23	7 (30.43)	0	0
	8-10	129	10 (7.75)	9 (6.98)	3 (2.33)
	11-13	421	51 (12.11)	25 (5.94)	15 (3.56)
	14-16	229	25 (10.92)	9 (3.93)	5 (2.18)

Among females, (n = 39, 10.80%) had light infections, (n = 16, 4.43%) moderate, and (n = 7, 1.94%) heavy. Among males, (n = 54, 12.24%) had light infections, (n = 27, 6.12%) moderate, and (n = 16, 3.63%) heavy. The 5–7 age group had the highest proportion of light infections (n = 7, 30.43%) and no moderate or heavy infections. The 8–10 age group had (n = 10, 7.75%) light, (n = 9, 6.98%) moderate, and (n = 3, 2.33%) heavy infections. The 11–13 age group had the highest proportions of moderate (n = 25, 5.94%) and heavy infections (n = 15, 3.56%), with (n = 51, 12.11%) light infections. The 14–16 age group had (n = 25, 10.92%) light, (n = 9, 3.93%) moderate, and (n = 5, 2.18%) heavy infections (Table 4).

### Logistic regression analysis of risk factors for *Schistosoma mansoni* Infection

Chi-square test and binary logistic regression analyses identified 10 variables associated with *S. mansoni* infection (i.e., had a p value less than 0.05) including respondent’s class, district, sub county, schools, water source for bathing, water source for washing, distance of water source from home/schools, water contact activities, father’s education, history of treatment with praziquantel three months before (Table 4). These variables were further analysed by backward stepwise multivariable logistic regression (Table 5). Backward stepwise multivariable logistic regression identified several independent risk factors significantly associated with *Schistosoma mansoni* infection among primary school children in the Lango sub-region (Table 5).

**Table 5:** A backward stepwise multivariable logistic regression analyses of the risk factors associated with *Schistosoma mansoni* infection among primary school children in Lango sub region.

Variable		B	Wald	Sig.	OR	95% C.I. for OR	
						Lower	Upper
	Class of students/grades of students		31.375	.000			
	P.1	.534	1.458	.227	1.705	.717	4.055
	P.2	.096	.037	.848	1.101	.414	2.928
	**P.3**	**1.159**	**12.344**	**.000**	**3.187**	**1.669**	**6.084**
	**P.4**	**1.170**	**13.079**	**.000**	**3.222**	**1.709**	**6.075**
	P.5	.253	.547	.460	1.288	.658	2.522
	**P.6**	**.964**	**6.616**	**.010**	**2.622**	**1.258**	**5.467**
	School of participants		89.843	.000			
	**Aberdyangotoo**	**1.572**	**5.758**	**.016**	**4.819**	**1.334**	**17.407**
	Abilonino	-.399	.239	.625	.671	.136	3.314
	Abolet	.019	.002	.967	1.020	.405	2.570
	**Acutkumu**	**-1.485**	**10.310**	**.001**	**.226**	**.091**	**.561**
	Akano	-.410	.777	.378	.664	.267	1.651
	Akore	-.360	.580	.446	.698	.276	1.761
	**Apedi**	**1.681**	**6.872**	**.009**	**5.373**	**1.529**	**18.889**
	**Ayara**	**3.557**	**27.588**	**.000**	**35.047**	**9.295**	**132.143**
	Ayer	.939	2.009	.156	2.556	.698	9.362
	**Bala**	**1.345**	**4.427**	**.035**	**3.837**	**1.097**	**13.425**
	Lwala	.709	1.049	.306	2.031	.523	7.884
	**Obot**	**-1.932**	**12.839**	**.000**	**.145**	**.050**	**.417**
	Obuto	-.669	.536	.464	.512	.085	3.074
	**Ocamoyang**	**-1.272**	**7.284**	**.007**	**.280**	**.111**	**.706**
	**Ogur**	**-1.399**	**7.763**	**.005**	**.247**	**.092**	**.660**
	Oketkwer	-.809	2.975	.085	.445	.178	1.117
	Okwerodot	1.016	2.292	.130	2.762	.741	10.291
	District		24.007	.000			
	**Kole**	**-.778**	**23.019**	**.000**	**.459**	**.334**	**.631**
	Lira City	-.733	2.744	.098	.481	.202	1.144
	**History of treatment with praziquantel 3 months ago (Yes)**	**-2.276**	**4.880**	**.027**	**.103**	**.014**	**.774**

NS not significant.

OR odd ratio.

CI confidence interval.

Significant results are bold.

The likelihood of infection varied significantly across grade levels (Wald = 31.375, *p* < 0.001). Compared with the reference group (P.7), children in P.3 (OR = 3.19, 95% CI: 1.67–6.08, *p* < 0.001), P.4 (OR = 3.22, 95% CI: 1.71–6.08, *p* < 0.001), and P.6 (OR = 2.62, 95% CI: 1.26–5.47, *p* = 0.010) had significantly higher odds of infection. Other grade levels (P.1, P.2, and P.5) showed no significant association.

Marked heterogeneity in infection risk was observed across schools (Wald = 89.843, *p* < 0.001). Pupils from Ayara Primary School had the highest risk, with an approximately 35-fold increased odds of infection compared to the reference group (OR = 35.05, 95% CI: 9.30–132.14, *p* < 0.001). Elevated risks were also observed in Apedi (OR = 5.37, 95% CI: 1.53–18.89, *p* = 0.009), Aberdyangotoo (OR = 4.82, 95% CI: 1.33–17.41, *p* = 0.016), and Bala (OR = 3.84, 95% CI: 1.10–13.43, *p* = 0.035) primary schools. In contrast, attending Acutkumu (OR = 0.23, 95% CI: 0.09–0.56, *p* = 0.001), Obot (OR = 0.15, 95% CI: 0.05–0.42, *p* < 0.001), Ocamoyang (OR = 0.28, 95% CI: 0.11–0.71, *p* = 0.007), and Ogur (OR = 0.25, 95% CI: 0.09–0.66, *p* = 0.005) primary schools was associated with significantly reduced odds of infection. No significant associations were found for children from Abilonino, Abolet, Akano, Akore, Obuto, Oketkwer, Okwerodot, Lwala, and Ayer schools.

District of residence was also a significant predictor (Wald = 24.007, *p* < 0.001). Pupils from Kole District were less likely to be infected than those from the reference district (Lira District), with a 54% reduction in odds (OR = 0.46, 95% CI: 0.33–0.63, *p* < 0.001). Lira City pupils had reduced odds as well, though this association was not statistically significant (OR = 0.48, 95% CI: 0.20–1.14, *p* = 0.098).

Finally, prior praziquantel treatment within the preceding 3 months was strongly protective (OR = 0.10, 95% CI: 0.01–0.77, *p* = 0.027), indicating that children who had received treatment were approximately 90% less likely to be infected at follow-up.

Overall, these findings highlight the strong influence of school-level and grade-specific factors, district of residence, and recent praziquantel treatment history on *S. mansoni* infection risk among schoolchildren in the Lango sub-region

## Discussion

The findings of this study highlight the significant burden of *Schistosoma mansoni* and other intestinal parasitic infections among primary school children in the Lango sub-region of Northern Uganda. The observed prevalence of (n = 277, 34.5%) for *S. mansoni* aligns with other studies conducted in endemic areas of Uganda [8] and sub-Saharan Africa [17], which report prevalence rates ranging from 20% to 60%, depending on geographical, environmental, and socio-economic factors [40–42].

### Prevalence and intensity of *Schistosoma mansoni* Infection

The high prevalence and intensity of *S. mansoni* infection in Lira District (n = 159, 57.4%), compared to Kole District (n = 110, 39.7%) and Lira City (n = 8, 2.9%), suggest localized transmission hotspots that require targeted interventions. The distribution of infections may be attributed to environmental factors, such as the presence of freshwater bodies, where children frequently engage in activities such as bathing, swimming, and fetching water. Prior studies indicate that open-water contact is a major risk factor for schistosomiasis transmission, particularly in regions where hygienic water sources are scarce [14,43–45]. The lower prevalence in Lira City may reflect better access to piped water, improved sanitation, and urbanization-related factors that reduce exposure risks. The observed infection intensities, light (n = 93, 11.6%), moderate (n = 43, 5.4%), and heavy (n = 23, 2.9%) underscore the ongoing transmission of schistosomiasis in this population. Similar studies have reported variations in infection intensities based on factors such as age, gender, and previous treatment history [46–50]. The presence of moderate and heavy infections indicates a high parasite burden, necessitating urgent deworming interventions and reinforcement of preventive strategies.

### Risk factors associated with schistosomiasis

Risk of infection differed significantly across grades and schools. Children in P.3 (OR = 3.19, 95% CI: 1.67–6.08, *p* < 0.001), P.4 (OR = 3.22, 95% CI: 1.71–6.08, *p* < 0.001), and P.6 (OR = 2.62, 95% CI: 1.26–5.47, *p* = 0.010) were more than twice as likely to be infected compared to P.7, suggesting that middle primary years coincide with increased water contact and behavioral exposure [14,17,43]. Additionally, at the school level, pupils from Ayara had the highest risk, with an approximately 35-fold increased odds of infection compared to the reference group (OR = 35.05, 95% CI: 9.30–132.14, *p* < 0.001). Similarly, Apedi (OR = 5.37, 95% CI: 1.53–18.89, *p* = 0.009), Aberdyangotoo (OR = 4.82, 95% CI: 1.33–17.41, *p* = 0.016), and Bala (OR = 3.84, 95% CI: 1.10–13.43, *p* = 0.035) schools had markedly higher risks. In contrast, attending Acutkumu (OR = 0.23, 95% CI: 0.09–0.56, *p* = 0.001), Obot (OR = 0.15, 95% CI: 0.05–0.42, *p* < 0.001), Ocamoyang (OR = 0.28, 95% CI: 0.11–0.71, *p* = 0.007), and Ogur (OR = 0.25, 95% CI: 0.09–0.66, *p* = 0.005) schools was associated with significantly reduced odds of infection. No significant associations were found for children from Abilonino, Abolet, Akano, Akore, Obuto, Oketkwer, Okwerodot, Lwala, and Ayer schools. This clustering reflects the micro-geographical nature of transmission, likely influenced by proximity to snail habitats, variations in local sanitation, and differences in exposure patterns [12,13,22,44]. Such heterogeneity underscores the need for fine-scale mapping and targeted interventions.

Furthermore, the District of residence was an important predictor of infection. Pupils from Kole District were less likely to be infected than those from the reference district (Lira District), with a 54% reduction in odds (OR = 0.46, 95% CI: 0.33–0.63, *p* < 0.001). Lira City pupils had reduced odds as well, though this association was not statistically significant (OR = 0.48, 95% CI: 0.20–1.14, *p* = 0.098). These variations may reflect environmental differences in exposure sites, urbanization effects, and disparities in access to safe water and sanitation [9,20,23]. Such findings highlight the need for geographically tailored control strategies.

Children who reported receiving praziquantel within the past three months was strongly protective (OR = 0.10, 95% CI: 0.01–0.77, *p* = 0.027), and were about 90% less likely to be infected, reinforcing the effectiveness of mass drug administration (MDA). However, the persistence of infections despite ongoing MDA points to gaps in coverage, compliance, and reinfection following treatment. This pattern mirrors findings from other endemic regions, where reliance on chemotherapy alone has been insufficient to interrupt transmission [51–55]. Our findings emphasize the need for strengthening drug distribution and coupling MDA with water, sanitation, and hygiene (WASH) improvements and health education [48,56,57].

Although the study area is included in Uganda’s national annual MDA program for schistosomiasis control, the implementation has been irregular, with variations in timing and coverage across schools and communities [57,58]. This may explain why the majority of children surveyed had not received praziquantel within the last three months, as shown in Table 3. Such inconsistencies in MDA implementation could contribute to persistent transmission and reinfection, emphasizing the need for more effective and consistent drug distribution strategies. Unfortunately, recent funding cuts by the United States government could have substantial implications for schistosomiasis prevention programs in Uganda. Many of these programs, including MDA initiatives, rely on international funding to sustain drug procurement, distribution, and community sensitization efforts [59]. A reduction in funding may lead to irregular drug administration, reduced treatment coverage, and weakened surveillance efforts, potentially reversing gains made in schistosomiasis control. Future research and policy efforts should explore alternative funding mechanisms to ensure the sustainability of prevention programs and mitigate potential increases in prevalence and reinfection rates.

Paternal education was inversely associated with infection risk, reflecting the broader influence of socioeconomic status and health literacy on schistosomiasis transmission. Families with higher education levels may have improved knowledge of preventive practices, greater access to clean water, and higher compliance with treatment programs. These results reaffirm the role of social determinants in shaping infection risk and control outcomes [60–65].

### Co-infection with other parasites

The study also identified the presence of co-infection with other parasites, with *Entamoeba coli* (n = 94, 11.7%) and *Ascaris lumbricoides* (n = 93, 11.6%) being the most prevalent. The occurrence of STHs, including *Hookworms* (n = 51, 6.4%) and *Trichuris trichiura* (n = 12, 1.5%), further highlights the burden of NTDs in the study area [51,66]. The co-existence of schistosomiasis and STHs has been reported in several regions, particularly where poor sanitation and hygiene (WASH) conditions prevail [7,67]. Such co-infections contribute to malnutrition, anemia, and cognitive impairments, necessitating integrated control strategies that address both waterborne and soil-transmitted parasitic infections [46]. The presence of rare infections, such as *Fasciola species* (n = 11, 1.4%) and *Taenia species* (n = 7, 0.9%), suggests possible zoonotic transmission, likely influenced by livestock interactions and consumption of contaminated food or water [49]. These findings emphasize the need for a One Health approach to disease control, incorporating human, animal, and environmental health interventions [56,68].

## Limitations of the study

A limitation of this study is that the logistic regression analysis did not explicitly control for potential confounders, such as socioeconomic status and access to sanitation facilities. While our analysis identified independent predictors of *S. mansoni* infection, residual confounding may still be present. Future studies should incorporate multivariable models that adjust for these factors to better isolate the independent effects of risk factors on schistosomiasis infection. We also acknowledge that the replacement of most initially selected Lira city schools following CHD + MDA resulted in a smaller Lira city sample and reduced precision for city-specific estimates. While our study primarily focused on *S. mansoni*, we also examined the prevalence of soil-transmitted helminths (STHs). However, due to limited data on intensity and risk factors for STHs, these aspects were not extensively analyzed. We acknowledge this as a limitation and suggest that future studies further explore the burden and determinants of STH infections in the study area. Finally, we used the Odongo-Aginya modification of the Kato Katz thick smear technique, which was developed and evaluated mainly for detecting *Schistosoma mansoni* and other intestinal helminths but has not been formally validated for intestinal protozoa. Therefore, the prevalence of protozoa such as *Giardia lamblia* and *Blastocystis spp*. in our study is most likely underestimated.

## Conclusion

This study demonstrates that *Schistosoma mansoni* and intestinal parasitic infections remain a significant public health concern among school-aged children in the Lango sub-region of northern Uganda. The high prevalence and moderate intensity of infection, coupled with marked heterogeneity across schools and districts, underscore the focal nature of transmission. Key risk factors included grade level, schools of attendance, district of residence, and low paternal education, while recent praziquantel treatment was strongly protective. These findings highlight that mass drug administration alone is insufficient to break transmission cycles. Sustainable control will require integrated strategies that combine consistent and equitable MDA delivery with targeted interventions for high-risk schools, improved water, sanitation, and hygiene (WASH) infrastructure, and strengthened community health education. Addressing socioeconomic disparities and ensuring continuity of funding are equally vital. Together, such context-specific and multisectoral approaches can reduce reinfection, alleviate morbidity, and accelerate progress toward the national and global goals of schistosomiasis elimination.

## Supporting information

S1 File Final dataset used in the analysis. (XSLX)

S2 File Questionnaire form for the study participants showing demographic information for school children, WASH, and socioeconomic factors. (PDF)
