# Supplementary material for: Prevalence, intensity, and risk factors of schistosomiasis and intestinal parasitic infections among primary school children in northern Uganda: Implications for public health interventions
Source: PLoS Negl Trop Dis. 2025 Dec 17;19(12):e0013827. doi: 10.1371/journal.pntd.0013827 (PMC12711080; doi:10.1371/journal.pntd.0013827)
Supplement: S2 File — (PDF) [file pntd.0013827.s002.pdf]

S2: Questionnaire form for study participants

(Tick or fill in where applicable)

A. DEMOGRAPHIC INFORMATION FOR SCHOOL CHLIDREN ONLY

a. Identification number …………….

Interviewer name ………………….

Interview date …………………

Gender Male ☐ Female ☐

Age in years ………..

Class ………………..

School …………………

Sub county…...…………………

Parish…………………………..

Village…………..……………………

b. Water, sanitation and hygiene practices

Water source for drinking: - Dam ☐ Bore-hole ☐ spring ☐ well ☐

Piped water ☐ Rain water ☐ Streams ☐

Water source for bathing ……………………

Water source for washing …………………...

c. Distance of water source from home/ school

< 500m ☐ 500-1000m ☐ >1000m ☐

d. Child's swimming habit in pond, dam or stream

Yes ☐ No ☐

e. Child's swimming frequency per week

Never (0) ☐ Moderate (1-3) ☐ High (4-7) ☐

h Child's swimming period in a day

7am-10am ☐ 11am-2pm ☐ 3pm- 6pm ☐

I Contact activities with stream or pond water

Bathing ☐ Laundry ☐ Fetching water ☐ Fishing ☐ Farming ☐

B Level of education of parents

Mother: None ☐ primary ☐ Secondary ☐ Tertiary ☐ University ☐

Father: None ☐ primary ☐ Secondary ☐ Tertiary ☐ University ☐

C Occupation of the parents

Mother: House wife ☐ farmer ☐ causal labourer ☐ salary earner ☐

Father: Business ☐ Farmer ☐ Petty trader ☐ Employed ☐

D Family income per month;

< 100000 UGx ☐ 100000- 200000 UGx ☐ > 200000UGx ☐

DEMOGRAPHY FOR OTHER COMMUNITY STUDY PARTICIPANTS

Identification number …………….

Interview date …………………….

a Occupation

Employed ☐ Self-employe☐ Far☐r Car wa☐r ☐ Herdsman ☐

Unemploye☐ Fisherman Others specify……………...

b Level of education

None ☐ Primary ☐ Secondary ☐ Tertiary University ☐

c Distance of home from open water source,

< 500m ☐ 500-1000m ☐ > 1000m ☐

d Source of domestic water;

Piped water ☐ Dam ☐ Stream ☐ Pond☐ Well ☐ Spring☐ Rainwater ☐

e History of contact with open water source

Yes ☐ No ☐

f Reason for contact with open water source

Crossing ☐ Washing ☐ Bathing ☐ Swimming ☐ Wetland farming☐Fetching water ☐

g Frequency of contact with other per week;

1-3 times ☐ more than 3 times ☐

h Duration of contact per day;

< 1 hour ☐ 1-2 hours ☐ > 3 hours ☐

I Family monthly income;

<100000UGx ☐ 100000-200000UGx ☐ > 200000UGxx ☐

J History of treatment with praziquantel in the last 3 months;

Yes ☐ No ☐
